# Fetuin‐A aggravates lipotoxicity in podocytes via interleukin‐1 signaling

**DOI:** 10.14814/phy2.13287

**Published:** 2017-05-29

**Authors:** Jana M. Orellana, Kapil Kampe, Friederike Schulze, Jonas Sieber, Andreas W. Jehle

**Affiliations:** ^1^ Department of Biomedicine, Molecular Nephrology University Hospital Basel Switzerland; ^2^ Department of Biomedicine, Diabetes Research University Hospital Basel Switzerland; ^3^ Harvard Medical School and Division of Nephrology Brigham and Women's Hospital Boston Massachusetts USA; ^4^ Department of Internal Medicine, Transplantation Immunology and Nephrology University Hospital Basel Switzerland

**Keywords:** Diabetic nephropathy, Fetuin‐A, free fatty acids, interleukin‐1, palmitic acid, toll‐like receptor

## Abstract

Sterile inflammation is considered critical in the pathogenesis of diabetic nephropathy (DN). Here we show that Fetuin‐A (FetA) or lipopolysaccharide (LPS) exacerbate palmitic acid‐induced podocyte death, which is associated with a strong induction of monocyte chemoattractant protein‐1 (MCP‐1) and keratinocyte chemoattractant (KC). Moreover, blockage of TLR4 prevents MCP‐1 and KC secretion and attenuates podocyte death induced by palmitic acid alone or combined with FetA. In addition, inhibition of interleukin‐1 (IL‐1) signaling by anakinra, a recombinant human IL‐1Ra, or a murinized anti‐IL‐1*β* antibody attenuates the inflammatory and ultimate cell death response elicited by FetA alone or combined with palmitic acid. In vivo short‐term therapy of diabetic DBA/2J mice with an anti‐IL1‐*β* antibody for 4 weeks prevented an increase in serum FetA and considerably decreased urinary tumor necrosis alpha (TNF‐*α*), a known risk factor for DN progression. In summary, our results suggest that FetA similarly to LPS leads to an inflammatory response in podocytes, which exacerbates palmitic acid‐induced podocyte death and our data imply a critical role for IL‐1*β* signaling in this process. The study offers the rational for prolonged in vivo studies aimed at testing anti‐IL‐1*β* therapy for prevention and treatment of DN.

## Introduction

Diabetic nephropathy (DN) has become a leading cause of end‐stage renal disease in industrialized countries, and most affected patients have type 2 diabetes (USRDS, 2003; Locatelli et al. 2004). Over the last few years it has become evident that sterile inflammation plays a central role in the pathogenesis of DN (Lim and Tesch 2012). Accumulation of macrophages was demonstrated in humans and rodent models of DN (Chow et al. 2004; Nguyen et al. 2006), and inhibition of inflammatory cell recruitment into the kidney protects from experimental DN (Chow et al. 2005; Awad et al. 2011). In addition to immune cells, intrinsic renal cells, such as podocytes and mesangial cells, can secrete proinflammatory cytokines, which may contribute to the inflammatory process and aggravate DN (Tesch et al. 1997; Sayyed et al. 2009).

Interleukin‐1*β* (IL‐1*β*) is a master regulator of inflammation in various tissues (Dinarello 2009). Intrinsic renal cells including podocytes are sources of IL‐1*β* in experimental models of glomerulonephritis (Niemir et al. 1997). The potential role of IL‐1*β* in the pathogenesis of DN dates back to 1996 when it was reported that a low expressing allele of the IL‐1 receptor antagonist (IL‐1Ra), which implies increased IL‐1 signaling, is associated with DN (Blakemore et al. 1996). More recently, it was reported that IL‐1*β* is elevated in plasma and in renal cortex extracts at the onset of DN, and anakinra, a recombinant human IL‐1Ra, can prevent or even reverse DN in different mouse models (Shahzad et al. 2015).

Toll‐like receptors (TLRs) are considered to play a key role in the inflammatory response underlying diabetes and its complications (Lin and Tang 2014). TLRs recognize pathogen‐associated molecular patterns such as lipopolysaccharide (LPS), and viral or bacterial nucleic acids (Akira et al. 2001). Activation of TLRs leads to a recruitment of adaptor proteins, which subsequently trigger downstream signaling cascades resulting in activation of nuclear factor‐*κ*B (NF‐*κ*B) (Akira et al. 2001). The transcription factor NF‐*κ*B induces a wide range of cytokines including IL‐1*β* (Hiscott et al. 1993), and monocyte chemoattractant protein‐1 (MCP‐1) (Ueda et al. 1994). TLRs are not only activated by pathogen‐associated molecular patterns, but also by endogenous danger signals released during tissue injury or metabolic stress (Akira et al. 2001).

Type 2 diabetes mellitus is characterized by hyperglycemia and dyslipidemia with increased levels of free fatty acids (FFAs) (Randle et al. 1963). Over the past years, evidence has accumulated that FFAs can induce the production of proinflammatory cytokines through activation of TLR4 and/or TLR2 (Lee et al. 2001; Shi et al. 2006; Boni‐Schnetzler et al. 2009). However, the mechanism how FFAs activate TLRs is under debate and current evidence suggests that FFAs do not directly bind to TLR4 (Erridge and Samani 2009). Recently, it was suggested that Fetuin‐A (FetA) may act as an endogenous ligand and molecular linker for FFAs to TLR4 (Pal et al. 2012). FetA is a liver‐derived, abundant plasma protein with multiple biological functions. FetA is known as a major carrier protein of FFAs in the circulation (Cayatte et al. 1990). As fetal bovine serum (FBS) contains about 20 mg/mL FetA, cell culture media with 10% FBS contain a considerable amount of FetA and therefore may explain previous reports suggesting that FFAs directly activate TLR4 (Pal et al. 2012). Importantly, studies targeting TLR4 genetically or pharmacologically revealed renoprotective effects in various murine models of DN (Kuwabara et al. 2012; Cha et al. 2013).

In the pathogenesis of DN, podocyte injury and loss are critical events (Wolf et al. 2005) and they precede albuminuria (Pagtalunan et al. 1997; Meyer et al. 1999; Dalla Vestra et al. 2003). Previously, we reported that podocytes are highly susceptible to the saturated FFA palmitic acid leading to podocyte death (Sieber et al. 2010). Mechanistically, palmitic acid‐induced podocyte death is linked to endoplasmic reticulum stress (Sieber et al. 2010), and its toxicity can be attenuated by monounsaturated FFAs by upregulation of stearoyl‐CoA desaturase 1 (Sieber et al. 2013), an enzyme converting saturated to monounsaturated FFAs, or by stimulation of fatty acid oxidation (Kampe et al. 2014). Whether inflammation contributes to palmitic acid‐induced podocyte death is currently unknown.

The objective of the present study was to investigate whether palmitic acid, FetA, or their combination elicits an inflammatory response in podocytes and whether they modify podocyte survival. In addition, we explored the potential role of TLR4 and IL‐1 signaling in these processes. In complementary in vivo studies, the short‐term effect of a murinized anti‐IL‐1 antibody was tested on serum FetA as well as on surrogate markers of DN.

## Materials and Methods

### Animal and experimental protocol

All experimental procedures were performed in accordance with and approved by the Swiss veterinary law and institutional guidelines. Eight‐week‐old DBA/2J (DBA) male mice were purchased from Charles River (Sulzfeld, Germany). Mice were maintained in 12‐h light/12‐h dark cycle, and provided food and water ad libitum. Mice were allowed to acclimatize to the animal facility for 1 week prior to experiment initiation.

### Induction of diabetes and anti‐IL‐1β treatment

Diabetes was induced in 8‐week‐old DBA mice by daily intraperitoneal (i.p.) injections of 40 mg/kg of streptozotocin (STZ; Sigma‐Aldrich, St. Louis, MO) for five consecutive days, freshly prepared in 50 mmol/L sodium citrate buffer at pH 4.5. Control mice were injected with sodium citrate buffer alone. One week after the last STZ injection, hyperglycemic mice (glucose ≥ 14 mmol/L) were fed a high‐fat diet (HFD) for 4 weeks. HFD was purchased from ssniff GmbH, Germany, containing 60% of fat (lard) (catalog no. E15742‐34). Anti‐IL‐1*β* antibody was kindly provided by Novartis (Basel, Switzerland), under a signed material transfer agreement (2015). Anti‐IL‐1*β* antibody was injected i.p. weekly at 10 mg/kg of body weight for the first 2 weeks and at 5 mg/kg for the following 2 weeks. Vehicle (saline solution) was injected to the control group.

### Measurement of blood glucose, serum FetA, and urinary TNF‐*α* and albumin levels

Fasting blood glucose was determined with a Glucometer (Freestyle; Abbott Diabetes Care, Inc., Alameda, CA) from tail vein blood of 6 h fasted mice. Serum FetA and urinary tumor necrosis alpha (TNF‐*α*) were measured using Duoset ELISA kit (R&D Systems) and mesoscale assays (Mesoscale Discovery, Rockville, MD) according to the manufacturer's instructions. Urinary albumin levels were determined using the albuminuria kit from Exocell (Philadelphia).

### Materials

Palmitic acid (P9767), low endotoxin and fatty acid free‐bovine serum albumin (BSA) (A8806), LPS (L2630), and IL‐1*β* (I5271) were purchased from Sigma (St. Louis, MO). Recombinant murine interferon‐gamma (CTK‐358‐2PS) was from MoBiTec (Goettingen, Germany). Type‐1 collagen was from BD Biosciences. Annexin V (A23204) and propidium iodide (P3566) were from Invitrogen (Eugene, Oregon).

**Figure 1:**
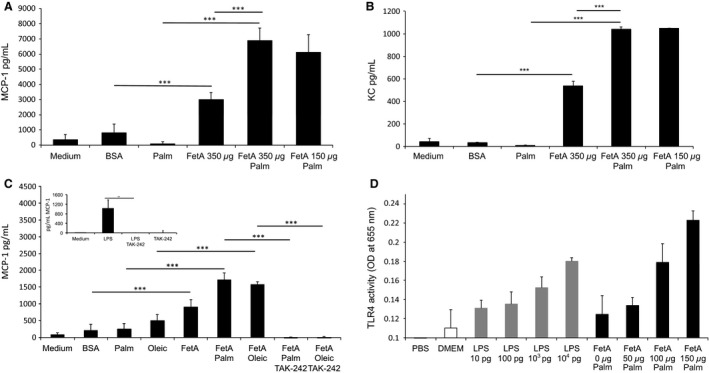
FetA but not palmitic acid or oleic acid stimulates MCP‐1 or KC secretion in podocyte. (A) Graph shows MCP‐1 expression after treating podocytes with 75 *μ*mol/L palmitic acid (palm) or bovine serum albumin (BSA) (control) either with or without 150–350 *μ*g/mL bovine FetA. Bar graph represents mean ± SD MCP‐1 levels (in pg/mL) in the supernatant after 12 h (*n* = 6, ***P < 0.001). (B) Graph shows KC expression after treating podocytes with 75 *μ*mol/L palm or BSA (control) either with or without 150–350 *μ*g/mL bovine FetA. Bar graph represents mean ± SD KC levels (in pg/mL) in the supernatant after 12 h (*n* = 6, ***P < 0.001). (C) Graph shows MCP‐1 expression in podocytes treated with 75 *μ*g/mL palm or 75 *μ*g/mL oleic acid (oleic) alone or in combination with 200 *μ*g/mL murine FetA. Bar graph represents mean ± SD MCP‐1 levels (in pg/mL) in the supernatant after 16 h of treatment and 1 h of preincubation with TAK‐242 (*n* = 4, ***P < 0.001). *Insert*: LPS induces MCP‐1 release, and TAK‐242 prevents chemokine expression. Podocytes were treated with 5 ng/mL LPS. Bar graph represents mean ± SD MCP‐1 levels (in pg/mL) in the supernatant after 16 h of treatment and 1 h of preincubation with TAK‐242 (*n* = 4, **P < 0.01). (D) Graph shows TLR4 activation in HEK‐Blue™ hTLR4 cells treated with LPS (10 pg, 100 pg/mL, 10^3^ pg/mL, 10^4^ pg/mL) or 150 *μ*mol/L palmitic acid in the presence of murine FetA (0 *μ*g/mL, 50 *μ*g/mL, 100 *μ*g/mL, 150 *μ*g/mL) for 18 h. Bar graph represents mean percentages ± SD of OD reading (*n* = 3).

Anakinra (Kineret^®^) was purchased from SOBI (Swedish Orphan Biovitrum AB). TAK‐242 (tlrl‐cli95) was from InvivoGen (Distributed by LabForce AG, Switzerland). Murinized Anti‐IL‐1*β* Ab is a noncommercialized product provided by Novartis. Bovine FetA was kindly provided by Prof. Jahnen‐Dechent, Aachen University, Germany, and the levels of endotoxins were tested in his laboratory with Endosafe ultrasensitive PTS, Charles River. Murine FetA was donated by Prof. Bhattacharya, Visva‐Bharati University, West Bengal, India. Experiments shown in Figure 1C and D were performed with murine FetA. All other experiments were done with bovine FetA.

### Cell culture, FFA preparation, and apoptosis assay

Murine podocytes were cultured as described previously (Sieber et al. 2010). Podocytes were differentiated for at least 11 days before starting experiments. All experiments were carried out in six‐well plates except for isolating protein or RNA for which 10 cm dishes were used. FFA preparations were done as described previously. The final concentration of palmitic acid was 200 *μ*mol/L complexed to BSA (0.2%), if not otherwise stated. Annexin V and PI stainings were performed as reported earlier (Sieber et al. 2010). Flow cytometry was carried out with CyAn™ ADP Analyzer (Beckman Coulter) and 20,000 cells were counted. Data from flow cytometry were analyzed using FLOWJO (Tree Star, Inc., Ashland, OR) software. Annexin V‐positive/PI‐negative podocytes were considered apoptotic, whereas Annexin V‐positive/PI‐positive podocytes were considered (late apoptotic) necrotic cells.

### Removal and measurement of endotoxins

FFA preparations, BSA, and bovine and murine FetA were depleted from potential endotoxin contaminations with EndoTrap^®^ HD (800063, Hyglos GmbH). Samples were passed four times through Endotrap columns. Endotoxin concentrations were subsequently measured with ToxinSensor Chromogenic LAL Endotoxin Assay Kit (L00350C, GenScript) according to the manufacturer's instructions if not otherwise indicated. All reactions were strictly performed at a constant temperature of 37°C in a heating block. Tips and plastic ware were endotoxin free. During experimental conditions cells were exposed to endotoxin concentrations of ≤ 0.15 EU/mL (≤ 0.015 ng/mL).

### TLR4 activation assay

TLR4 activation assay was done by using HEK‐Blue hTLR4 reporter cells containing TLR4/NF‐*κ*B/SEAP purchased from InvivoGen, CA, USA. Cells were cultured in 75 cm^2^ flask with DMEM (#41965, Invitrogen) media including 4.5 g/L glucose, 4 mmol/L l‐glutamine, 10% FBS, penicillin/streptomycin, and Normocin according to manufacturer's instructions. For experiments involving TLR4 activation, HEK‐Blue™ detection media without FBS was used. Experiments were done in 96‐well plates with 150 *μ*mol/L palmitic acid in the presence of FetA ranging from 0 to 150 *μ*g/mL or LPS from 10 to 10^4^ pg/mL for 6 h. Secreted SEAP downstream of TLR4 activation induced a color reaction of the cell culture media (purple to blue) and the absorbance was quantified with a Synergy H1 hybrid reader (BioTek) at 640 nm.

### Measurement of cytokine and chemokine expression

Differentiated podocytes were cultured in 75 cm^2^ flasks and were re‐seeded in 96‐well plates for 24 h, at 6000 cells/well. All experiments were performed under serum starvation conditions (RPMI‐1640, with 0.2% heat‐inactivated FBS and 5 mmol/L glucose) including a 16‐h preincubation to synchronize the cells. MCP1 and KC expression was quantified using Duoset ELISA kit according to manufacturer's protocol (DY479 for MCP‐1; DY453 for KC, R&D Systems purchased from Zug, Switzerland).

### Statistical analysis

All experiments were performed at least 4 times if not otherwise indicated. Data are expressed as means ± SD. Analysis of variance (ANOVA) and Bonferroni *t*‐tests were used for statistical analysis using GraphPad Prism 6 software.

## Results

### FetA but not palmitic acid or oleic acid stimulate MCP‐1 or KC secretion in podocytes

To investigate whether palmitic acid leads to an inflammatory response in podocytes, we measured MCP‐1 and KC in the cell culture media of podocytes after incubation with 75 *μ*mol/L palmitic acid (complexed to BSA) for 12 h. As shown in Figure 1A and B, palmitic acid did not stimulate the secretion of these cytokines compared to the control media with BSA alone. As it has been suggested that FetA may be necessary to elicit an inflammatory response by palmitic acid (Pal et al. 2012), podocytes were incubated with FetA or FetA in combination with palmitic acid (Fig. 1A and B). In contrast to palmitic acid, FetA significantly stimulated MCP‐1 and KC secretion by 368 ± 57% (*P* < 0.001) and 1729 ± 135% (*P* < 0.001), respectively. The combination of FetA and palmitic acid led to a further increase in MCP‐1 and KC by 129 ± 28% (*P* < 0.001) and 94 ± 3% (*P* < 0.001), respectively, compared to FetA alone. As shown in Figure 1C, similar results were obtained with oleic acid, which itself was not able to stimulate MCP‐1 secretion significantly. However, FetA in combination with oleic acid increased MCP‐1 by 73 ± 10% (*P* < 0.0001) compared to FetA.

As the proinflammatory response of FetA is reported to involve TLR4 (Pal et al. 2012), we inhibited TLR4 signaling with TAK‐242 (resatorvid), a specific small‐molecule inhibitor of TLR4 signaling (Matsunaga et al. 2011). TAK‐242 completely prevented the induced MCP‐1 secretion by the prototypic TLR4 ligand LPS (Fig. 1C, insert) as well as by FetA in combination with palmitic or oleic acid (Fig. 1C).

To further investigate the potential role of FetA in stimulating TLR‐4 signaling, we took advantage of a commercially available TLR4 reporter HEK cell line. As shown in Figure 1D, increasing concentrations of FetA from 50 to 150 *μ*g/mL in the presence of a constant concentration of 150 *μ*mol/L palmitic acid dose dependently stimulated TLR4 similar to the activation by LPS in the range from 10 pg to 10 ng/mL.

### FetA moderately aggravates and inhibition of TLR4 signaling attenuates palmitic acid‐induced podocyte death

**Figure 2:**
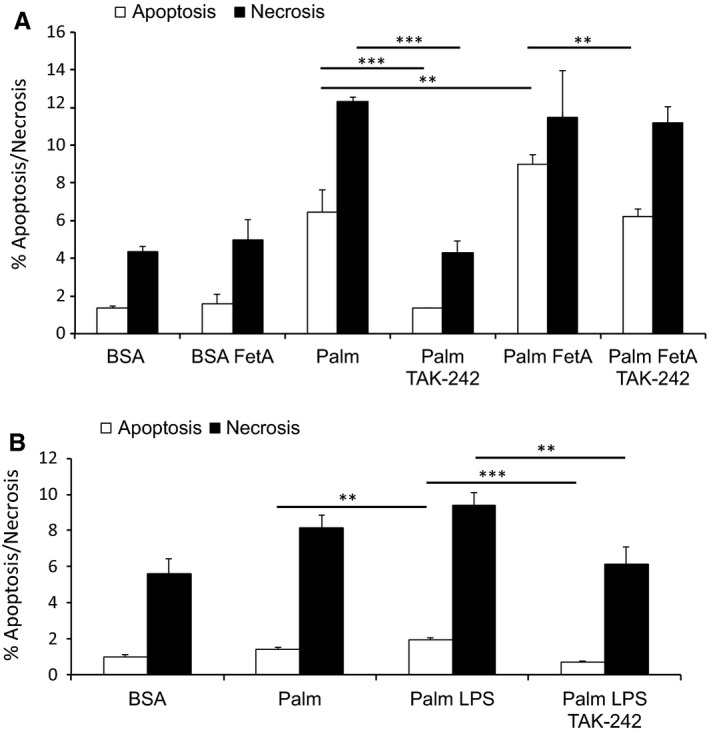
TLR4 blockage attenuates bovine FetA or LPS exacerbated palmitic acid‐induced podocyte death. (A) Graph shows podocytes treated with 200 *μ*mol/L palmitic acid (palm) or bovine serum albumin (BSA) (control) alone or in combination with 200 *μ*g/mL bovine FetA for 48 h and preincubated with 1 ng/mL TAK‐242 for 1 h. Bar graph represents the mean percentages ± SD of Annexin V‐positive/PI‐negative (early apoptotic) and Annexin V‐positive/PI‐positive (late apoptotic/necrotic) podocytes (*n* = 3, **P < 0.01, ***P < 0.001). (B) Graph shows podocytes exposed to BSA (control), 200 *μ*mol/L palm alone or combined with 5 ng/mL LPS for 48 h and with or without preincubation of 1 ng/mL TAK‐242 for 1 h. Bar graph represents the mean percentages ± SD of Annexin V‐positive/PI‐negative (early apoptotic) and Annexin V‐positive/PI‐positive (late apoptotic/necrotic) podocytes (*n* = 3, **P < 0.01, ***P < 0.001).

To investigate whether FetA modulates cell death induced by palmitic acid, podocytes were treated with FetA alone or in combination with 200 *μ*mol/L palmitic acid for 48 h. As shown in Figure 2A, FetA had no effect on podocyte viability, but moderately exacerbated palmitic acid‐induced podocyte death. Specifically, FetA further increased apoptosis in podocytes by 21 ± 0% (*P* < 0.05). An effect on necrotic podocytes was not consistently observed, most likely due to higher variations and the previously reported observation that necrotic podocytes disintegrate, which ultimately leads to underestimation of necrotic cells (Sieber et al. 2013). Similarly, LPS had no visible effect on podocyte necrosis, but increased palmitic acid‐induced apoptosis by 29 ± 7% (*P* < 0.001) (Fig. 2B). TAK‐242 prevented the FetA‐induced increase in apoptotic podocytes (Fig. 2A) as well as the increase induced by LPS (Fig. 2B). Surprisingly, compared to podocytes treated with palmitic acid alone, the presence of TAK‐242 decreased palmitic acid‐induced apoptosis by 79 ± 1% (*P* < 0.001) and necrosis by 65 ± 5% (*P* < 0.001) (Fig. 2A). This unexpected finding may indicate modulation of podocyte death by a constitutive activation of TLR4 or additional protective off‐target effects.

### Anakinra and an anti‐IL‐1β antibody attenuate MCP‐1 expression induced by FetA and palmitic acid

**Figure 3:**
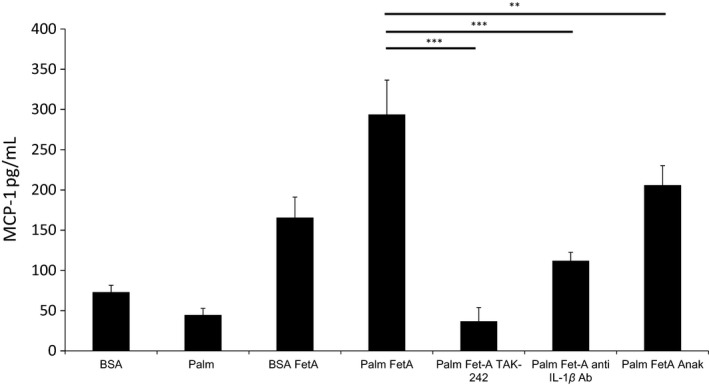
The TLR4 blocker TAK‐242 or IL‐1 neutralization prevent chemokine expression induced by bovine FetA alone or in combination with FFAs. Podocytes were treated with 75 *μ*mol/L palm or BSA (control) alone or in combination with 200 *μ*g/mL bovine FetA, and preincubated with 1 ng/mL TLR4 blocker (TAK‐242) or 1 *μ*g/mL IL‐1R antagonist (anakinra) or 3.3 *μ*g/mL anti‐IL‐1*β* antibody (anti‐IL‐1*β* Ab). Bar graphs represent mean ± SD MCP‐1 levels (in pg/mL) in the supernatant after 1 h of preincubation and treatment for 16 h (*n* = 3, *P < 0.05, **P < 0.01, ***P < 0.001).

To investigate the potential involvement of IL‐1*β* on FetA and palmitic acid‐induced MCP‐1 secretion, podocytes were cotreated in the presence or absence of the recombinant human IL‐1Ra antagonist anakinra and a murinized neutralizing anti‐IL‐1*β* antibody for 16 h. As shown in Figure 3, the anti‐IL‐1*β* antibody and anakinra reduced MCP‐1 secretion by 53 ± 17% (*P* < 0.001) and by 30 ± 9% (*P* < 0.001), respectively.

### Anakinra and anti‐IL‐1*β* antibody attenuate podocyte death induced by palmitic acid and FetA

**Figure 4:**
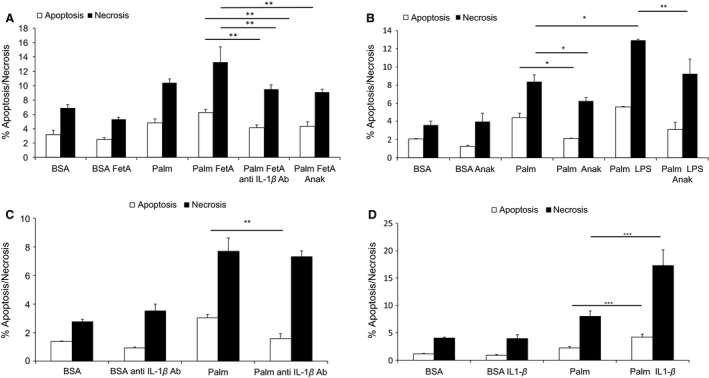
Antagonization of IL‐1 attenuates FetA or LPS exacerbated palmitic acid‐induced podocyte death. (A) Graph shows podocytes treated with 200 *μ*mol/L palmitic acid (palm) or bovine serum albumin (BSA) (control) alone or in combination with 200 *μ*g/mL bovine FetA for 48 h, and preincubated for 1 h with 1 ng/mL TAK‐242, or 3.3 *μ*g/mL anti‐IL‐1*β* antibody, or 1 *μ*g/mL IL‐1Ra (Anakinra). Bar graph represents the mean percentages ± SD of Annexin V‐positive/PI‐negative (early apoptotic) and Annexin V‐positive/PI‐positive (late apoptotic/necrotic) podocytes (*n* = 3, **P < 0.01, ***P < 0.001). (B) Graph shows podocytes exposed to palm or BSA (control) alone or in combination with 1 ng/mL LPS, and preincubated for 30 min with 1 *μ*g/mL anakinra. Podocytes were treated for 48 h. Bar graph represents the mean percentages ± SD of Annexin V‐positive/PI‐negative (early apoptotic) and Annexin V‐positive/PI‐positive (late apoptotic/necrotic) podocytes (*n* = 3, *P < 0.05, **P < 0.01). (C) Graph represents podocytes exposed to 200 *μ*mol/L palm or BSA (control) alone or preincubated for 1 h with 3.3 *μ*g/mL anti‐IL1*β* antibody. Podocytes were treated for 48 h. Bar graph represents the mean percentages ± SD of Annexin V‐positive/PI‐negative (early apoptotic) and Annexin V‐positive/PI‐positive (late apoptotic/necrotic) podocytes (*n* = 3 *P < 0.05, **P < 0.01). (D) Graph shows podocytes treated with 200 *μ*mol/L palm or BSA (control) and 5 ng/mL IL‐1*β* for 48 h. Bar graph represents the mean percentages ± SD of Annexin V‐positive/PI‐negative (early apoptotic) and Annexin V‐positive/PI‐positive (late apoptotic/necrotic) podocytes (*n* = 3, ***P < 0.001).

To examine whether IL‐1 signaling plays a role in the aggravating effect of FetA on palmitic acid‐induced cell death, podocytes were preincubated for 1 h with anti‐IL‐1*β* or anakinra prior to the addition of palmitic acid or the combination of palmitic acid and FetA for 48 h. Both the anti‐IL‐1*β* antibody and anakinra significantly reduced apoptosis by 34 ± 6% (*P* < 0.05) and 30 ± 6% (*P* < 0.05), and necrosis by 29 ± 5% (*P* < 0.05) and 33 ± 3% (*P* < 0.01), respectively (Fig. 4A).

Anakinra also partially prevented the exacerbating effect of LPS on palmitic acid‐induced apoptosis and necrosis by 45 ± 14% (*P* < 0.01) and 23 ± 13%, respectively (Fig. 4B). Interestingly, the anti‐IL‐1*β* antibody and anakinra also attenuated podocyte death induced by palmitic acid alone (Fig. 4B and C). Finally, we investigated whether IL‐1*β* induces podocyte death. IL‐1*β* alone in a dose range from 5 to 20 ng/mL did not affect the viability of podocytes (data not shown); however, 5 ng/mL IL‐1*β* significantly exacerbated palmitic acid‐induced podocyte apoptosis and necrosis by 117 ± 35% (*P* < 0.001) and 90 ± 24% (*P* < 0.001), respectively (Fig. 4D).

### Anti‐IL‐1β treatment reduces serum FetA and urinary TNF‐α levels in diabetic mice

**Figure 5:**
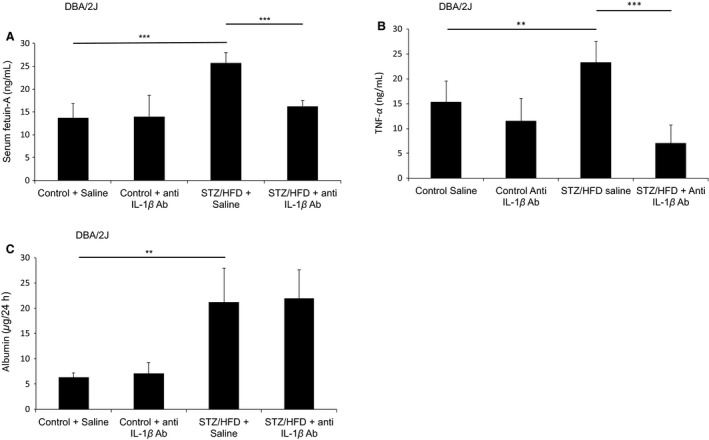
Anti‐IL‐1*β* treatment reduces serum FetA and urinary TNF‐*α* levels in diabetic DBA/2J mice. (A) One week after the last streptozotocin (STZ) injection, DBA/2J mice were fed a HFD and control groups were maintained on chow diet for 4 weeks. Anti‐IL‐1*β* antibody treatment was started simultaneously as mice were fed a HFD and administered i.p. at 10 *μ*g/g of mice for the first 2 weeks of treatment and then maintained at 5 *μ*g/g for another 2 weeks. Bar graph represents mean (SD) of serum FetA levels (*n* = 6, ***P < 0.001). (B) Bar graph represents mean (SD) of urinary TNF‐*α* levels (*n* = 6, **P < 0.01, ***P < 0. 001). (C) Bar graph represents mean (SD) of urinary albumin values collected in 24 h (*n* = 6, **P < 0.01).

To complement our in vitro findings, we performed a pilot study to investigate the effect of the anti‐IL‐1*β* antibody in diabetic DBA/2J mice. Diabetes was induced with multiple low‐dose STZ injections, and to further promote a renal phenotype, mice were fed a high‐fat diet (HFD) similar to previous studies (Wang et al. 2009). The metabolic characteristics of the DBA/2J mice are depicted in Table 1. At the end of the study, diabetic mice showed threefold higher average fasting blood glucose levels, which were not affected by anti‐IL‐1*β* treatment. However, the diabetes‐associated elevation of serum FetA levels (88 ± 16%, *P* < 0.001) was significantly attenuated by anti‐IL‐1*β* antibody 63 ± 5% (*P* < 0.001; Fig. 5A).

**Table 1:** Metabolic characteristic of DBA/2J mice experiments

	Control + Saline	Control + Anti‐IL‐1*β* Ab	STZ (HFD) + Saline	STZ (HFD) + Anti‐IL‐1β Ab
Age (weeks) at the start of experiment or induction of diabetes	9	9	9	9
Weight at induction of diabetes (g)	26.5 ± 0.96	26.5 ± 1.82	25.8 ± 1.53	25.7 ± 1.35
Weight at the end of experiment (g)	33.3 ± 2.35	33.3 ± 2.85	24.8 ± 2.06	25.5 ± 1.67
Fasting blood glucose (mmol) at 10 days after STZ injection	5.5 ± 0.88	5.6 ± 0.27	11.2 ± 2.55	12.0 ± 2.48
Fasting glucose (mmol) at the end of experiment	5.8 ± 1.72	5.4 ± 0.86	17.4 ± 2.06	18.0 ± 2.04

As previous studies linked an increase in urinary TNF‐*α* levels to the progression of albuminuria (Kalantarinia et al. 2003; Wu et al. 2013), we investigated whether treatment with the anti‐IL‐1*β* antibody affects urinary TNF‐*α* levels in diabetic DBA/2J mice. Indeed, diabetic mice showed increased urine TNF‐*α* levels (69 ± 19%, *P* < 0.01) compared to nondiabetic controls. Intriguingly, diabetic mice treated with the anti‐IL‐1*β* antibody revealed 71 ± 20% (*P* < 0.001) lower TNF‐*α* levels in the urine (Fig. 5B). To address whether the treatment has any renoprotective effects, urinary albumin levels were determined as a marker for kidney filter integrity. Even though diabetic DBA/2J mice had elevated albumin levels in the urine (299 ± 74%, *P* < 0.01; Fig. 5C) after a follow‐up of 4 weeks, not unexpectedly the levels were only moderately increased. Therefore, future long‐term studies have to address whether neutralizing IL‐1*β* can prevent the progression of DN. However, these findings indicate that a therapy targeting IL‐1*β* can prevent the diabetes‐associated increase in urine TNF‐*α* levels and serum FetA, which are risk factors for the development of DN.

## Discussion

In the present study, we demonstrate that FetA aggravates palmitic acid‐induced podocyte death. This effect is associated with an inflammatory response and involves IL‐1*β* signaling.

Several lines of evidence suggest that the deleterious effect of FetA may involve TLR4 signaling. Specifically, using genetically engineered HEK‐Blue hTLR4 cells as a reporter system FetA led to a dose‐dependent (range of 50–150 *μ*g/mL) TLR4 activation in the presence of 150 *μ*mol/L palmitic acid. Using the same assay, it was previously shown that FetA alone or combined with palmitic acid activates TLR4, though palmitic acid alone had no significant effect (Pal et al. 2012). A similar response was observed with the prototypical TLR4 ligand LPS. In contrast to FetA, the dose of LPS was increased logarithmically from 10 to 10^4^ pg/mL. This suggests that the mechanism of FetA on TLR4 may differ from LPS. To minimize interference with any residual endotoxin contamination, FetA as well as all FFA complexes were purified using polymyxin B columns. Of interest, the effect of FetA on the HEK‐Blue‐hTLR4 reporter cell line was only observed with murine but not with bovine FetA (data not shown) suggesting a species‐dependent and potentially a glycosylation‐dependent receptor activation (Pal et al. 2012). However, in podocytes both bovine and murine FetA significantly stimulated MCP‐1 release (Figs. 1A, C, and 3), which was further increased by palmitic acid. Consistent with the reporter assay data, the specific TLR4 inhibitor TAK‐242 (Matsunaga et al. 2011) prevented the MCP‐1 release in podocytes (Figs. 1C and 3) and our data suggest that the observed proinflammatory response involves TLR4 signaling.

Interestingly, and in contrast to previous studies using other cells including pancreatic *β*‐cells (Lee et al. 2001; Shi et al. 2006; Boni‐Schnetzler et al. 2009), no stimulation of MCP‐1 or KC could be observed in podocytes incubated with palmitic acid alone, although palmitic acid together with FetA exacerbated the inflammatory response. This may be explained by inherent differences of specific cell types. Alternatively, most cell culture studies are performed with 10% FBS, which contains about 20 mg/mL FetA (Pal et al. 2012). Previously, it has been suggested that FetA, FFAs, and TLR4 build a ternary complex (Pal et al. 2012) though part of the evidence for a direct interaction of FetA and TLR4 results from a yeast two‐hybrid assay and needs further explanation as glycosylation of FetA has been shown to be critical (Pal et al. 2012). Clearly, more experiments are needed to clarify the exact interplay between FetA, FFAs, and TLR4.

Of note, both FetA and LPS had no effect on podocyte survival, but exacerbated palmitic acid‐induced podocyte death, and this could be partially prevented by the TLR4 inhibitor TAK‐242 (Fig. 2A and B). The finding that LPS exacerbated palmitic acid‐induced podocyte death may at least in part explain albuminuria in septic patients (Basu et al. 2010) as bacterial endotoxins and FFAs are increased in this setting (Nogueira et al. 2008). Of note, endotoxin levels are also reported to be elevated in patients with metabolic syndrome and may contribute to the progression of DN (Lassenius et al. 2011).

Unexpectedly, TAK‐242 dramatically reduced palmitic acid‐induced podocyte death. Even though FetA, BSA, and FFAs complexed to BSA were purified from endotoxin with polymyxin B, stimulation of TLR4 by a residual endotoxin contamination, though unlikely, cannot be completely excluded. If so, the residual endotoxin concentration would be in the lower range of healthy human blood donors, reported to be between 1.00 and 0.01 EU/mL (Nadhazi et al. 2002). Alternatively, the protective effect of TAK‐242 on palmitic acid‐induced podocyte death can be explained by a constitutively active TLR4 in podocytes. Although TAK‐242 binds specifically to an intracellular domain of TLR4 (Matsunaga et al. 2011), we cannot rule out additional protective off‐target effects.

An interesting outcome of the current study was that inhibition of IL‐1 signaling by anakinra, a recombinant human IL‐1Ra, or a murinized anti‐IL‐1*β* antibody could attenuate the inflammatory response elicited by FetA and palmitic acid. In addition, anakinra and the anti‐IL‐1*β* antibody attenuated podocyte death induced by palmitic acid alone or in combination with FetA. This clearly indicates that palmitic acid‐induced podocyte death is partially mediated by IL‐1, even more specifically by IL‐1*β*. Interestingly, podocyte death could not be induced by IL‐1*β* alone, as it was previously reported for pancreatic beta‐cells (Maedler et al. 2004), however, IL‐1*β* was able to exacerbate palmitic acid‐induced podocyte death. Together this indicates that the proapoptotic effect of IL‐1*β* in podocytes depends on a second insult elicited by palmitic acid or by the combination of palmitic acid and FetA. Of interest, the protective effects of anakinra and the anti‐IL‐1*β* antibody on podocytes exposed to FetA and/or palmitic acid implies the presence of IL‐1*β*; however, IL‐1*β* levels in the cell culture media of treated podocytes were in the signal noise. This is a well‐known phenomenon as IL‐1*β* is highly active and levels are often under the detection limit of conventional assays and the involvement of IL‐1*β* can only be demonstrated indirectly, by its neutralization (Marianne Böni‐Schnetzler, pers. comm.).

In a pilot short‐term study, we investigated the effect of a murinized anti‐IL‐1*β* antibody in insulinopenic DBA/2J mice (STZ model) fed a high‐fat diet. Treatment with the anti‐IL‐1*β* antibody suppressed the increase in urinary TNF‐*α* levels observed in diabetic mice. This may be relevant as urinary TNF‐*α* is a risk factor for albuminuria (Kalantarinia et al. 2003), and TNF‐*α* is reported to induce podocyte death (Ryu et al. 2012; Tejada et al. 2008, and our unpublished data). Of importance, treatment with the anti‐IL 1*β* antibody prevented the increase in serum FetA levels. In this context, a community‐based study in China is noteworthy as people in the highest FetA tertile had a twofold increased risk for developing albuminuria over a follow‐up period of 4 years (Lv et al. 2016). This could potentially indicate that the lower serum FetA concentrations together with the reduced urinary TNF‐*α* levels observed in mice treated with the anti‐IL‐1*β* antibody may predict long‐term protection by the anti‐IL‐1*β* antibody against DN. Of note, urinary FetA levels were under the detection limit (data not shown), though in a human study in patients with type 2 diabetes urinary FetA was identified as a new risk factor for renal injury (Inoue et al. 2013). In this short‐term diabetes model, diabetic mice did not develop kidney pathologies. Even though urine albumin excretion is significantly increased in diabetic mice, this increase was very limited, and therefore, further in vivo studies with a longer follow‐up are needed to investigate the effect of the anti‐IL‐1*β* antibody for the prevention and treatment of DN. An apparent limitation of the current study is that we do not know whether STZ diabetes, HFD, or their combination lead to an increase in serum FetA and urinary TNF‐*α* which can be prevented by anti‐IL‐1*β* treatment, and additional control groups with STZ‐diabetes and HFD alone need to be included in future experiments. Furthermore, a recent study in mice deficient in NLRP3 (nucleotide‐binding domain and leucine‐rich repeat pyrin 3 domain) in nonmyeloid‐derived cells linked inflammasome formation, which activates caspase‐1, and thus maturation of IL‐1*β* (Kumar and Anders 2014), to the pathogenesis of DN (Shahzad et al. 2015). In addition, the same study reports that anakinra can protect from or even reverse the course DN in several mouse models (Shahzad et al. 2015).

In summary, our results suggest that FetA leads to an inflammatory response in podocytes, which is further enhanced by palmitic acid and exacerbates palmitic acid‐induced podocyte death. They underscore the role of TLR4 signaling in the proinflammatory effect of FetA. In addition, our data support a critical role for IL‐1 signaling in palmitic acid‐induced podocyte death. In vivo treatment with an anti‐IL‐1*β* antibody prevented an increase in serum FetA concentrations and suppressed urinary TNF‐*α* levels in diabetic mice. These findings offer the rationale for future studies addressing the long‐term effects of IL‐1*β* signaling inhibition on the development and course of DN.

## Conflict of Interest

The authors have no conflict of interests.
